# Thoracoscopic radical esophagectomy and laparoscopic transhiatal lymph node dissection for superficial esophageal cancer associated with lymph node metastases in the dorsal area of the thoracic aorta

**DOI:** 10.1186/s40792-015-0030-8

**Published:** 2015-03-10

**Authors:** Itasu Ninomiya, Koichi Okamoto, Tomoya Tsukada, Hiroto Saito, Sachio Fushida, Hiroko Ikeda, Tetsuo Ohta

**Affiliations:** Gastroenterologic Surgery, Department of Oncology, Division of Cancer Medicine, Graduate School of Medical Science, Kanazawa University, 13-1, Takaramachi, Kanazawa, Ishikawa 920-8641 Japan; Section of Diagnostic Pathology, Kanazawa University Hospital, Kanazawa, Ishikawa 920-8641 Japan

**Keywords:** Esophageal cancer, Lymphatic metastasis, Video-assisted surgery, Thoracic-aorta

## Abstract

Esophageal cancer invading the muscularis mucosa sometimes involves regional lymph node metastases. However, lymph node metastases are rare in the dorsal area of the thoracic aorta. We describe a patient with an intramucosal esophageal cancer invading the muscularis mucosa, accompanied by lymph node metastases in the dorsal area of the thoracic aorta. These lesions were successfully resected by hand-assisted laparoscopic surgery using a transhiatal approach. A 60-year-old man was diagnosed with superficial esophageal cancer during a routine health examination. Endoscopic examination and ultrasonography revealed a superficial cancer, of diameter 6.0 cm, invading the submucosal layer and intramural metastases caudal to the primary tumor. Enhanced computed tomography and F-deoxyglucose positron emission tomography demonstrated the two metastatic lymph nodes, one in the dorsal area of the thoracic aorta and the other near the left gastric artery. Thoracoscopic radical esophagectomy with three-field lymph node dissection was performed. The metastatic lymph node in the dorsal area of the thoracic aorta was successfully removed by hand-assisted laparoscopic surgery using a transhiatal approach. Histopathological examination showed primary cancer invading the muscularis mucosa and intramural metastases in the lamina propria mucosa and submucosal layer. The pathological diagnosis according to the Japanese classification of esophageal cancer was MtLt, 47 mm, 0-IIa + IIb, pT1a-MM, ie(+), INF-b, ly3, v0, pN4(4a), pIM1, M0, and pstage IVa. The patient underwent two courses of adjuvant chemotherapy, consisting of CDDP and 5-fluorouracil. At present, 1 year and 8 months after surgery, the patient remains alive without tumor recurrence. Although the lymph node in the dorsal area of the thoracic aorta is not recognized as regional nodes of thoracic esophageal cancer, solitary mediastinal metastases from a mucosal cancer may indicate the existence of direct lymphatic flow from the thoracic esophagus to the retroaortic region. Transhiatal approach by hand-assisted laparoscopic surgery is useful to dissect the metastatic lymph node in the dorsal area of the thoracic aorta.

## Background

Superficial esophageal cancer invading the muscularis mucosa (MM) may occasionally be associated with lymph node metastases [1,2]. In the Japanese classification of esophageal cancer, regional lymph nodes have been classified by their associations with patient prognosis, based on their rates of metastasis and patient survival [3,4]. Lymph node metastases are rare in the dorsal area of the thoracic aorta. Therefore, the lymph node in the dorsal area of the thoracic aorta is not recognized as regional nodes. We describe a patient with an intramucosal esophageal cancer invading the MM, accompanied by lymph node metastases in the dorsal area of the thoracic aorta. These lesions were successfully resected by hand-assisted laparoscopic surgery (HALS) using a transhiatal approach.

## Case presentation

**Figure 1:**
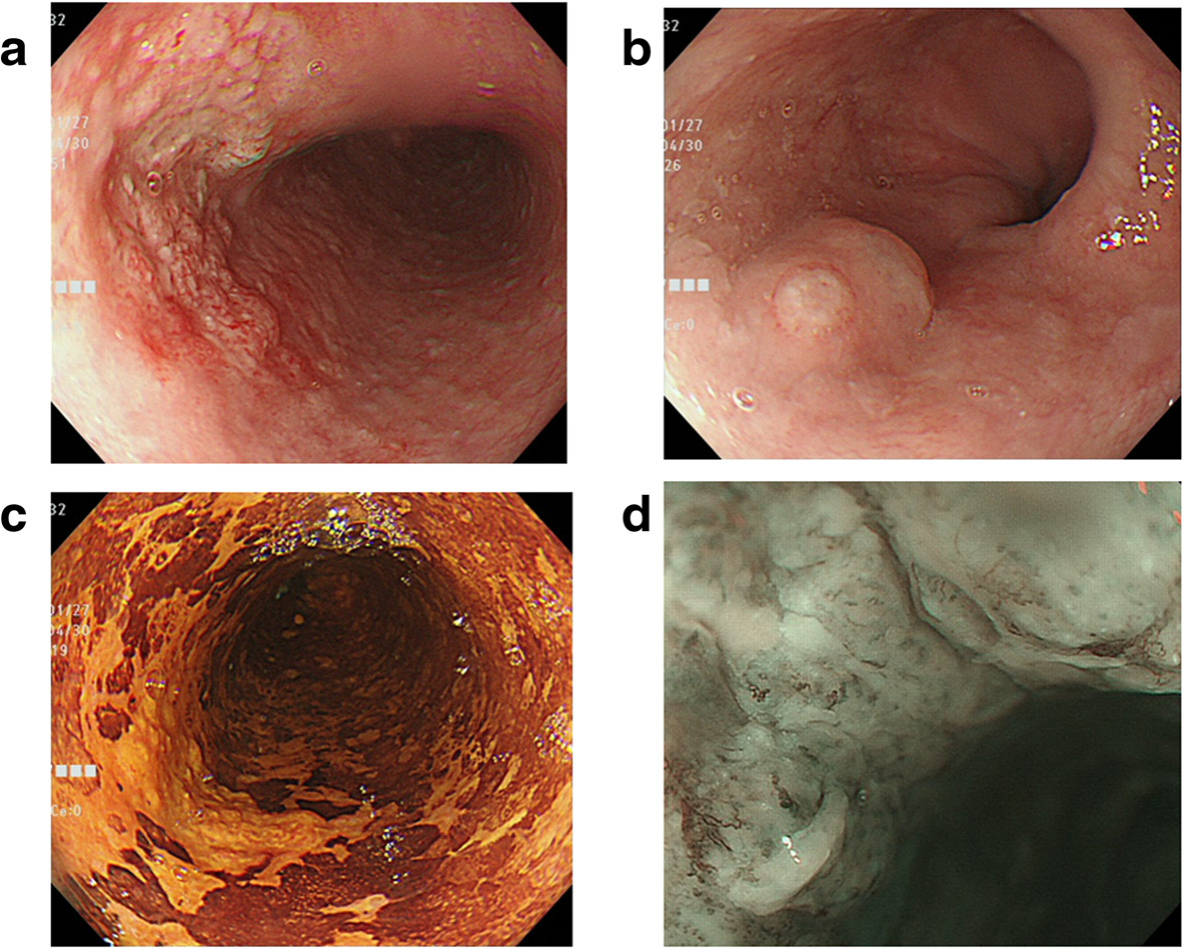
**The endoscopic findings.** Endoscopic findings of the primary esophageal tumor under white light **(a, b)**, iodine staining **(c)**, and narrow-band imaging **(d)**. **(a)** An irregular ulcerative tumor in the middle to lower thoracic esophagus. **(b)** An elevated lesion covered with normal epithelium on the anal side of the main tumor. Biopsy scar was present on the elevated lesion. **(c)** Iodine staining showing surface extension of the tumor. **(d)** Destroyed intrapapillary capillary loops under magnified narrow-band imaging.

**Figure 2:**
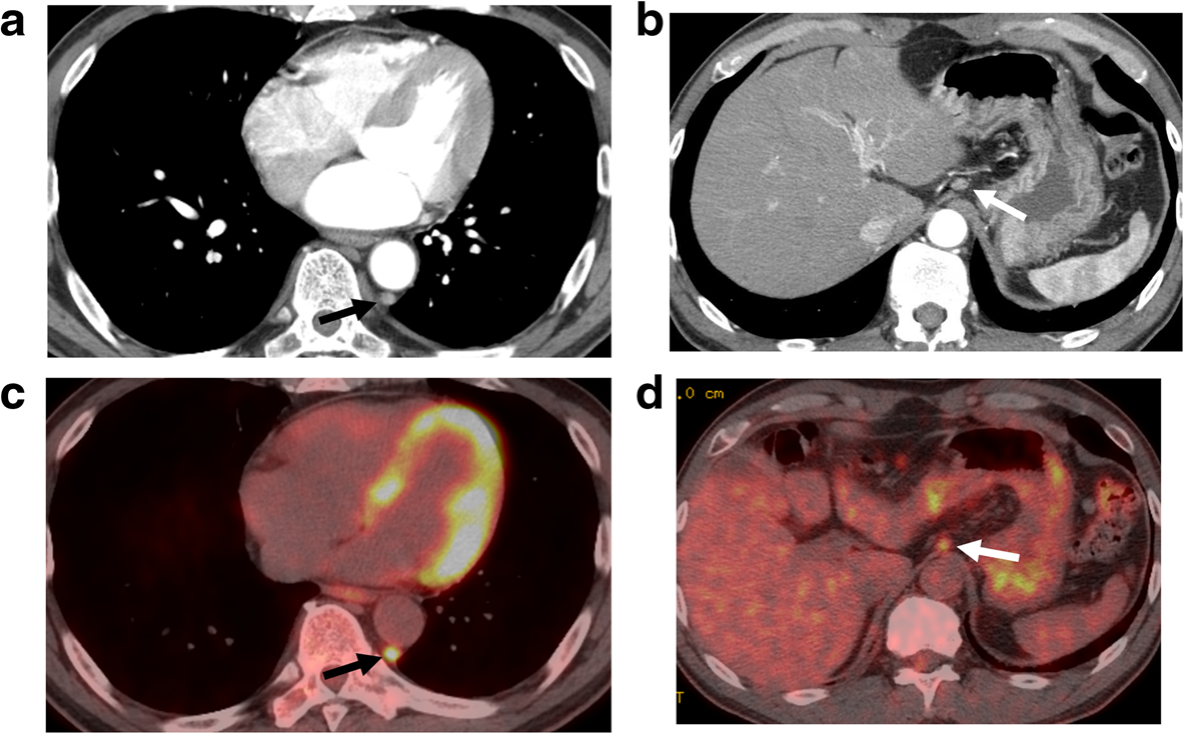
**The computed tomography findings.** Findings of the metastatic mediastinal **(a, c)** and abdominal **(b, d)** lymph nodes. Computed tomography (CT) showing a swollen lymph node, 0.8 cm in size, in the retroaortic area and accumulation of F-deoxyglucose (FDG) in FDG positron emission tomography (FDG-PET) (black arrow). CT scan and FDG-PET imaging also showed a swollen lymph node with FDG accumulation in the perigastric node along the left gastric artery (white arrow).

**Figure 3:**
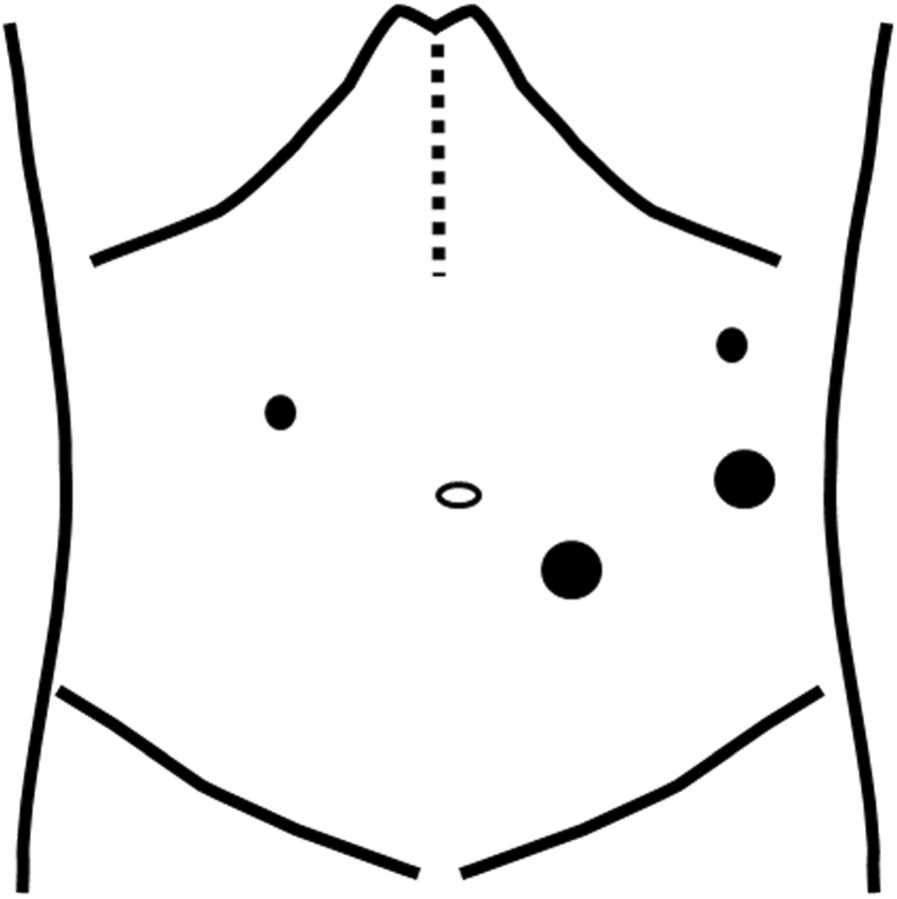
**Schematic illustration of the mini-laparotomyand the port sites in the hand-assisted laparoscopic surgery.** Small and large dots indicate the 5 mm and 12 mm ports, respectively. Dot line indicates the mini-laparotomy.

**Figure 4:**
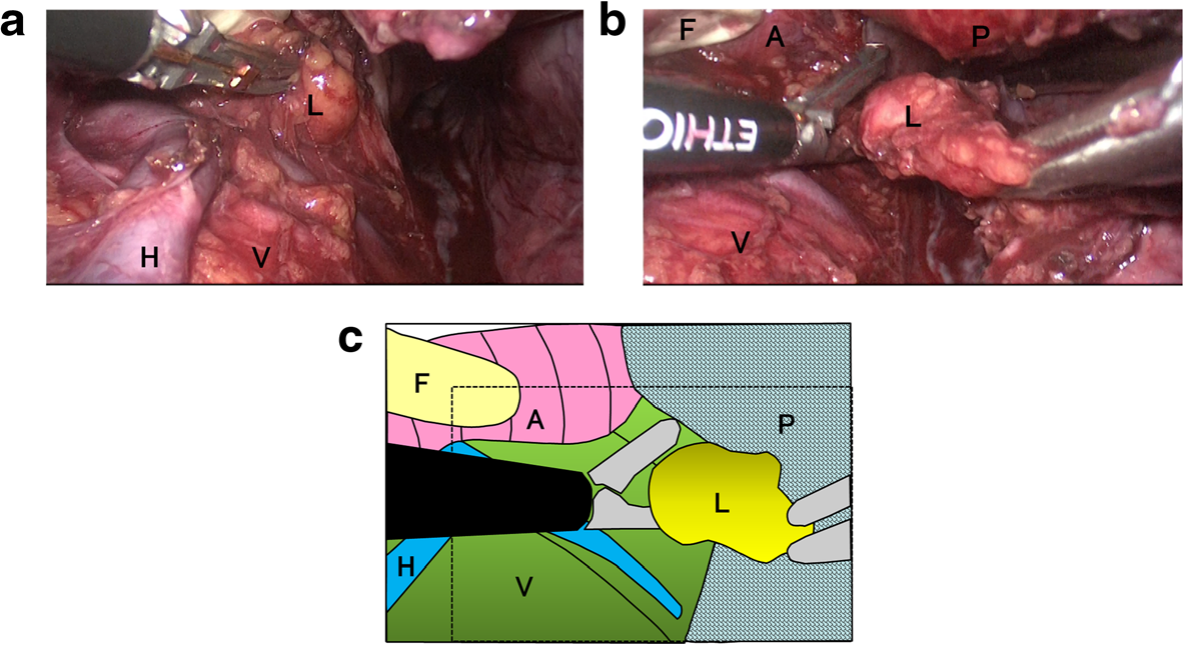
**Operative findings and schematic illustration of transhiatal dissection of the retroaortic lymph node.** The retroaortic space was visualized by anterior rotation of the thoracic aorta by hand-assisted laparoscopic surgery. **(a,** **b)** Dissection of the metastatic lymph node by EnSeal device. **(c)** Schematic illustration of Figure 4b. The area spaced by dotted line in Figure 4c corresponds to Figure 4b. A, aorta; F, finger of the operator; H, hemi-azygos vein; L, metastatic lymph node; P, parietal pleura; V, vertebra.

A 60-year-old man was diagnosed with esophageal cancer found incidentally on upper gastrointestinal endoscopy during a health examination. A superficial irregular ulcerative area was observed in the middle to lower third of the thoracic esophagus (Figure 1a), with an elevated lesion covered by normal epithelium found caudal to the main lesion (Figure 1b). A biopsy specimen of the latter obtained during the health examination was histologically shown to be a squamous cell carcinoma. Iodine staining showed that the lesion was about 6.0 cm in diameter and occupied two thirds of the esophageal lumen (Figure 1c). Narrow-band imaging showed highly destroyed intrapapillary capillary loops in the ulcerative area, indicating tumor invasion of the submucosal layer (Figure 1d). Endoscopic ultrasonography with a 20-MHz transducer estimated the depth of tumor invasion as cT1b (SM1). Histological examination of the biopsy specimen collected from the ulcerative lesion showed squamous cell carcinoma. Computed tomography scan could not detect the primary tumor in the esophagus, but detected an enhanced swollen lymph node, 0.8 cm in diameter, in the dorsal area of the thoracic aorta (Figure 2a), as well as a swollen lymph node along the left gastric artery (Figure 2b). F-deoxyglucose (FDG) positron emission tomography showed high FDG uptake by the esophageal tumor, as well as by the retroaortic (Figure 2c) and perigastric (Figure 2d) lymph nodes. These lymph nodes were suspected of being metastases of esophageal cancer. The patient was diagnosed with a superficial, esophageal squamous cell carcinoma in the middle and lower thoracic esophagus with intramural metastasis and perigastric and distant lymph node metastases, and was classified as having cT1bN4M0IM1 stage IVa according to the Japanese classification of esophageal cancer [3,4]. Although the recommended therapeutic strategy for stage IV disease is not surgery, we tried to resect all metastatic lymph nodes to confirm the cancer spread by histopathologic examination. The patient underwent video-assisted thoracoscopic esophagectomy in the left lateral position [5] with three-field lymph node dissection. HALS was used for all abdominal procedures. The retroaortic lymph node could not be identified by a thoracoscope inserted into the right thoracic cavity. The metastatic lymph node in the dorsal area of thoracic aorta was identified by mediastinal scope inserted from abdominal port and dissected by HALS using a transhiatal approach and a pneumomediastinum method [6]. Following thoracoscopic surgery for mediastinal lymph node dissection and esophageal transection in the upper mediastinum, the patient was placed in the supine position and underwent the HALS procedure. A 7-cm upper-abdominal median incision was created for insertion of the operator’s left hand. Four ports were inserted as shown in Figure 3. Carbon dioxide was introduced into the intra-abdominal space, and pressure in the pneumoperitoneum was controlled at 10 mmHg. After usual gastric mobilization and abdominal lymph node dissection, the esophagus was pulled down to the abdominal cavity from the esophageal hiatus. After enlargement of the esophageal hiatus, the adventitia of the thoracic aorta were exposed near the crura of the diaphragm, from the anterior to the left side and then to the dorsal side, in that order. Using pneumomediastinum and anterior retraction of thoracic aorta enabled visualization of the anatomy around the dorsal area of the thoracic aorta. A swollen lymph node between the dorsal side of the aorta and the hemiazygos vein was dissected, along with surrounding fatty tissue, using an EnSeal device (Ethicon, Cincinnati, OH, USA) without injuring the hemiazygos vein and intercostal arteries (Figure 4). A gastric conduit was created and raised through the posterior mediastinal route. The operation was completed by cervical esophagogastrostomy with circular stapling. The patient’s postoperative clinical course was uneventful, without postoperative bleeding, chylothorax, or anastomotic leakage. However, he experienced delayed, left recurrent laryngeal nerve palsy, which became apparent 1 week after surgery but disappeared 3 months later. The patient underwent two courses of adjuvant chemotherapy, consisting of CDDP and 5-fluorouracil. At present, 1 year and 8 months after surgery, the patient remains alive without tumor recurrence.

**Figure 5:**
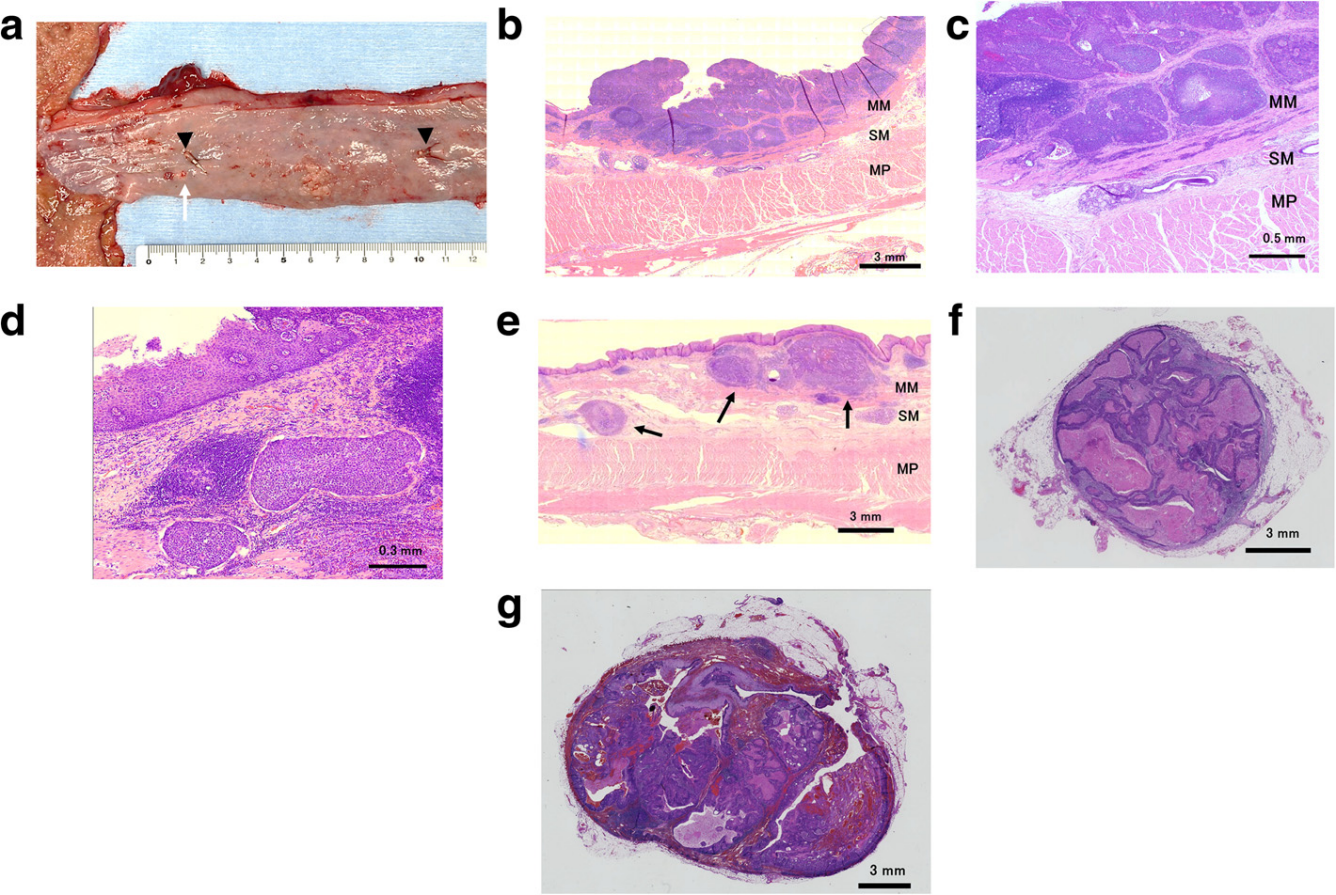
**Macroscopic and pathological findings of the resected specimen. (a)** Macroscopic findings. Marking clips were endoscopically attached before surgery to the oral and distal ends of the intraepithelial spread of the tumor (arrow head). The white arrow indicates the intramural metastasis. **(b**, **c)** Proliferation of the squamous cell carcinoma to the muscularis mucosa layer in the esophagus. **(d)** Lymphatic invasion of the primary tumor. **(e)** The intramural metastasis (arrows) was located primarily in the lamina propria mucosa and partly in the submucosal layer. **(f)** Metastatic lymph node retrieved from the dorsal area of the thoracic aorta. **(g)** Metastatic lymph node in the perigastric nodes along the left gastric artery. MM, muscularis mucosa; SM, submucosa; MP, muscularis propria.

Postoperative histopathological examination showed proliferation of the squamous cell carcinoma to the MM layer, with intraepithelial spread in the esophagus (Figure 5a,b,c). The primary tumor showed high lymphatic invasion (Figure 5d). The elevated lesion caudal to the primary tumor was found to be an intramural metastasis (Figure 5e), located primarily in the mucosal layer of the lamina propria and partly in the submucosal layer. Two lymph node metastases were found, one in the dorsal area of the thoracic aorta (0.8 cm in size, Figure 5f) and the other along the left gastric artery (2.0 cm in size, Figure 5g). Therefore, the pathological diagnosis was MtLt, 47 mm, 0-IIa + IIb, pT1a-MM, ie(+), INF-b, ly3, v0, pN4(4a), pIM1, M0, and pstage IVa.

## Discussion

The esophagus has multidirectional lymphatic flow, resulting in widespread and random patterns of lymph node metastasis from the cervical to the abdominal areas. Lymphatics form a dense submucosal plexus running longitudinally and non-segmentally, resulting in metastases to distant regional lymph nodes. The rate of metastasis and the importance of dissection of regional lymph nodes in patients with thoracic esophageal cancer were previously investigated in a large number of patients who underwent radical esophagectomy [7]. However, none of these patients had metastases in the retroaortic area. Recurrence after radical esophagectomy has been reported in the distant viscera, the locoregional area, and at multiple sites [8]. A report described two patients with solitary nodal recurrence in the dorsal area of the thoracic aorta after curative resection of esophageal cancer [9]. This area is therefore a site of tumor recurrence after curative surgery operation. In contrast, only two case reports have described primary esophageal cancer associated with lymph node metastases in the dorsal area of the thoracic aorta [10,11]. Horio et al. [10] speculated that metastases in the dorsal area of the thoracic aorta may indicate nearby extensive lymph-node metastasis, even if the metastasis appears solitary on preoperative examination. In our institution, lymphatic recurrence after radical thoracoscopic esophagectomy was observed in 23 of 146 (15.8%) patients. Nodal recurrence in the mediastinal area is infrequent after radical thoracoscopic esophagectomy [5]. However, six (4.1%) patients showed lymphatic recurrence in the dorsal area of the thoracic aorta. Among these six patients, three patients survived after local therapy by radiation or surgical resection. Therefore, we convinced surgical resection to the lymph node metastasis in the dorsal area of the thoracic aorta might have a certain degree of survival benefit in some cases.

Previous studies showed 1% to 8.5% of mucosal esophageal squamous cell carcinoma showed lymph node metastasis [12-15]. Multicenter retrospective cohort study showed only two patients (1.9%) among 104 patients with 111 lesions of esophageal squamous cell cancer invading the MM developed lymph node metastasis after endoscopic mucosal resection [2]. However, lymph node metastasis was observed in 18.0% in patients with esophageal squamous cell cancer invading the MM who underwent radical esophagectomy with lymph node dissection [16]. Lymphatic permeation has been reported to be a good predictor of lymph node metastasis in patients with superficial esophageal cancer [17]. As far as we know, there have been no reports of the mucosal esophageal squamous cell cancer accompanied by distant lymph node metastasis. The primary tumor in our patient had highly aggressive metastatic potential, as indicated by lymphatic invasion of the primary tumor, the intramural metastasis, and lymph node metastases in two separate areas, the distant mediastinal and abdominal regions. However, the primary tumor was a mucosal cancer invading the MM layer, with only one metastatic lymph node in the mediastinum. Solitary mediastinal metastases from a mucosal cancer may indicate the existence of direct lymphatic flow from the thoracic esophagus to the retroaortic region.

Previously, lymph node metastases in the dorsal area of the thoracic aorta were removed using a transthoracic approach from the left thoracic cavity [10,11]. To remove all the regional lymph nodes as well as the metastatic retroaortic lymph node by transthoracic approach, a bilateral transthoracic procedure is needed in this case. We used a transhiatal approach to dissect the lymph node in the dorsal area of the thoracic aorta of our patient. The use of pneumomediastinum and anterior retraction of the thoracic aorta made visualization of the retroaortic area possible. In performing transhiatal approach for mediastinal dissection, counter retraction to enlarge the esophageal hiatus is necessary. We created four ports to insert the retractors, an Enseal device (Ethicon Endo-Surgery, Inc., Ohio, USA) and a scope in HALS procedure.

We could safely dissect the metastatic lymph node using an EnSeal device and complete curative dissection without using a left transthoracic approach, thus minimizing surgical trauma.

## Conclusions

In conclusion, the lymph nodes in the dorsal area of the thoracic aorta may be metastatic sites in patients with thoracic esophageal cancer. A laparoscopic transhiatal approach may be useful in dissecting lymph nodes in the dorsal area of the thoracic aorta in circumstances where lymph node metastasis in this area is suspected.

## Consent

Written informed consent was obtained from the patient for publication of this Case report and any accompanying images.
